# Neglected mycobiome in HIV infection: Alterations, common fungal diseases and antifungal immunity

**DOI:** 10.3389/fimmu.2022.1015775

**Published:** 2022-11-10

**Authors:** Shuang Li, Xiaodong Yang, Christiane Moog, Hao Wu, Bin Su, Tong Zhang

**Affiliations:** ^1^ Beijing Key Laboratory for HIV/AIDS Research, Clinical and Research Center for Infectious Diseases, Beijing Youan Hospital, Capital Medical University, Beijing, China; ^2^ Sino-French Joint Laboratory for Research on Humoral Immune Response to HIV Infection, Clinical and Research Center for Infectious Diseases, Beijing Youan Hospital, Capital Medical University, Beijing, China; ^3^ Laboratoire d’ImmunoRhumatologie Moléculaire, Institut national de la santé et de la recherche médicale (INSERM) UMR_S 1109, Institut thématique interdisciplinaire (ITI) de Médecine de Précision de Strasbourg, Transplantex NG, Faculté de Médecine, Fédération Hospitalo-Universitaire OMICARE, Fédération de Médecine Translationnelle de Strasbourg (FMTS), Université de Strasbourg, Strasbourg, France; ^4^ Vaccine Research Institute (VRI), Créteil, France

**Keywords:** mycobiome, fungal diseases, fungal microbiota, antifungal immunity, HIV

## Abstract

Human immunodeficiency virus (HIV) infection might have effects on both the human bacteriome and mycobiome. Although many studies have focused on alteration of the bacteriome in HIV infection, only a handful of studies have also characterized the composition of the mycobiome in HIV-infected individuals. Studies have shown that compromised immunity in HIV infection might contribute to the development of opportunistic fungal infections. Despite effective antiretroviral therapy (ART), opportunistic fungal infections continue to be a major cause of HIV-related mortality. Human immune responses are known to play a critical role in controlling fungal infections. However, the effect of HIV infection on innate and adaptive antifungal immunity remains unclear. Here, we review recent advances in understanding of the fungal microbiota composition and common fungal diseases in the setting of HIV. Moreover, we discuss innate and adaptive antifungal immunity in HIV infection.

## Introduction

The human mycobiome inhabits the skin, respiratory tract, gastrointestinal tract, genitourinary tract, and other mucosal surfaces of the host. It has been shown that over 600 different fungi can cause disease in humans (1). In addition, over 300 million people are affected by serious fungal diseases, causing over 1.6 million deaths each year (2). The increased incidence of global epidemic mycoses might be attributed to changes in the environment, population growth in endemic areas, and increased human immunodeficiency virus (HIV)-related immunosuppressive status (3).

In HIV infection, differences in bacterial population composition, including the oral microbiome (4, 5), lung microbiome (6, 7) and gut microbiome (8, 9), have been reported in HIV-infected compared to uninfected individuals. In addition, immunity compromised by HIV infection may lead to altered fungal composition, promoting the development of opportunistic fungal infections in HIV-infected individuals (10). It has been shown that opportunistic fungal infections have an unacceptably high toll on people living with HIV (PLWH) and are a major driver of HIV-related death (11). Although ART might decrease the mortality rate, the substantial burden of fungal disease remains high for HIV-infected individuals who are undiagnosed, untreated or fail ART (12). Fungal diseases in HIV infection have also not received sufficient attention from the global community (11).

It has been shown that host innate and adaptive immune responses play an important role in controlling fungal infections (13). Innate antifungal immune responses are triggered when fungal antigens, such as α- and β-glucans, O- and N-linked mannans, and chitin, stimulate pattern recognition receptors (PRRs) expressed on host cells, including C-lectin receptors (CLRs), NOD (nucleotide-binding and oligomerization domain)-like receptors (NLRs), and Toll-like receptors (TLRs), to initiate signal transduction cascades, which promote the production of chemokines and cytokines to eliminate fungal pathogens and activate adaptive responses (14). However, the substantial loss of CD4^+^ T cells in HIV infection might lead to deficiencies in antifungal immunity, contributing to an increased risk of opportunistic fungal infections. Furthermore, depletion of interleukin (IL)-17 and IL-22-producing T helper (Th) 17 cells might result in impaired integrity of mucosal epithelial barriers, leading to fungal translocation from the gut lumen into the systemic circulation (15, 16). Microbial translocation might contribute to HIV-associated immune activation and inflammation, as well as the development of non-AIDS events (15, 16).

Because appropriate culture conditions remain unclear, most of the human mycobiome is nonculturable by culture-dependent methods (17). However, with the advent of new techniques, next-generation sequencing has been widely used for mycobiome detection in recent years (18). Internal transcribed spacer (ITS) sequencing and 18S rRNA are the most applied techniques to detect the mycobiome (19). In this review, we discuss alterations in the mycobiome and common fungal diseases in HIV infection, as well as the effects of HIV infection on innate and adaptive antifungal immunity.

## Alterations of the mycobiome in HIV infection

### The oral mycobiome in HIV infection

Alterations in oral bacterial communities and virome in HIV-infected individuals have been reported in many studies (4, 5, 20–22). The possible reasons for oral microbiome dysbiosis might be the disrupted oral immunity caused by HIV infection, including changes in secretory components in saliva, deficiency of innate immune responses and adaptive immune responses (5).

In addition to oral bacterial and virus communities, the oral mycobiome might contribute to understanding host−pathogen interactions that occur in HIV infection (23). For example, Ghannoum et al. characterized the oral mycobiome in healthy individuals using ITS sequencing (24), detecting 74 culturable and 11 nonculturable fungal genera (24). *Candida* species were the most frequent of the oral mycobiome (isolated from 75% of participants), followed by *Cladosporium* (65%), *Aureobasidium* and *Saccharomycetales* (50%, respectively) (24). Previous studies have demonstrated that oral fungal colonization is altered in HIV infection (25–28) (**Table 1**). In HIV-infected individuals, *Candida* (92%), *Epicoccum* (33%), and *Alternaria* (25%) are the most common genera in the oral mycobiome, whereas the most abundant oral mycobiome genera in HIV-uninfected controls are *Candida*, *Pichia*, and *Fusarium*, present in 58%, 33%, and 33%, respectively (25, 26). A recent study also compared the oral mycobiome between 30 HIV-infected individuals and 30 healthy controls and explored the effect of ART on the oral mycobiome in HIV infection. They found *Candida*, *Mortierella*, *Malassezia*, *Simplicillium*, and *Penicillium* to be significantly increased in HIV-infected individuals and dramatically decreased after ART. In contrast, the abundances of *Verticillium*, *Issatchenkia*, and *Alternaria* were significantly increased in PLWH after ART (27). They found that the composition of the oral mycobiome in the HIV-infected subjects after 6 months of ART was similar to that in the HIV-uninfected individuals. Moreover, *Mortierella*, *Malassezia*, *Simplicillium*, and *Chaetomium* were positively associated with viral load (VL), and *Verticillium*, *Thyrostroma* and *Archaeorhizomyces* were negatively associated with VL and positively correlated with CD4^+^ T-cell counts. In addition, *Saccharomyces* was positively correlated with VL and negatively associated with CD4^+^ T-cell counts (27). Therefore, HIV infection and ART administration might impact on the composition of the oral mycobiome, and the dysbiosis of oral mycobiome in HIV infection could be partially restored after ART. Furthermore, some oral fungi were sensitive to the changes in CD4^+^ T-cell counts and VL in the blood of HIV-infected individuals, thus changes in the oral mycobiome in HIV infection after ART may reflect the immune status of patients.

**Table 1:** The mycobiome in HIV infection.

References	Study cohort	Samples		Design		Mycobiome alterations
**The oral mycobiome in HIV infection**						
Chang et al. (27)	30 HIV^+^ subjects prior to and after 6 months of ART and 30 healthy controls	Saliva	Cross-sectional and longitudinal		• Increased *Candida*, *Mortierella*, *Malassezia*, *Simplicillium*, and *Penicillium* in the HIV group, decreasing after ART. • Increased *Verticillium*, *Issatchenkia*, and *Alternaria* in HIV^+^ subjects after ART.	
Fidel et al. (28)	149 HIV^+^ subjects and 88 HIV^-^ subjects	Oral rinse	Cross-sectional		• Predominated by four major clusters: *Candida albicans*, *Candida dubliniensis*, *Malassezia restricta*, and *Saccharomyces cerevisiae*. • Several clinical variables affect the oral mycobiome, including HIV positivity and ART.	
Mukherjee et al. (25)	12 HIV-infected and 12 uninfected individuals	Oral rinse	Cross-sectional		• Enrichment of *Candida*, *Epicoccum*, and *Alternaria* in HIV-infected individuals (present in 92%, 33%, and 25%, respectively) and *Candida*, *Pichia*, and *Fusarium* in uninfected individuals (58%, 33%, and 33%, respectively).	
**The respiratory tract mycobiome in HIV infection**						
Cui et al. (29)	32 HIV-infected and 24 HIV-uninfected individuals	Oral washes, induced sputa, and BAL		Cross-sectional		• Increased *Pneumocystis jirovecii*, *Junghuhnia nitida*, *Phlebia tremellosa*, *Oxyporus latemarginatus*, *Sebacina incrustans*, *Ceriporia lacerata*, *Pezizella discrete*, *Trametes hirsute*, and *Daedaleopsis confragosa* in HIV-infected individuals.
Bittinger et al. (30)	19 HIV^+^ subjects and 12 healthy controls	Oropharyngeal wash and BAL		Cross-sectional		• Increased clinical pathogens *Pneumocystis*, *Cryptococcus*, and *Aspergillus* in HIV-infected individuals.
**The gut mycobiome in HIV infection**						
Hamad et al. (31)	31 HIV-infected individuals and 12 uninfected-HIV individuals	Fecal		Cross-sectional		• Enriched *Ascomycota*, *Pichia*, *Penicillium brevicompactum* and *Penicillium* in healthy controls and enriched *Candida albicans* and *Candida tropicalis* in HIV-infected individuals.
Wu et al. (32)	75 HIV-infected patients and 55 HIV-uninfected participants	Fecal		Cross-sectional		• Nectriaceae, Hypocreales, and Sordariomycetes were the top 3 fungal taxa in HIV-infected individuals. While Basidiomycota, Phallaceae, and Phallales were particularly enriched in HIV-uninfected controls.
Yin et al. (33)	18 HIV-infected patients and 22 healthy controls	Fecal		Cross-sectional		• *Aspergillus* was the most abundant genus (49.92%) in the HIV-infected group, while the most abundant fungal genus was *Candida* (38.31%) in the healthy controls. • Unclassified_Aspergillaceae and Dirkmeia were enriched in the high-CD4^+^ T-cell group, while *Candida*, Sordariales, Saccharomycetaceae, and *Neocosmospora* were enriched in the low-CD4^+^ T-cell group.

HIV, human immunodeficiency virus; ART, antiretroviral therapy; BAL, bronchoalveolar lavage.

### The respiratory tract mycobiome in HIV infection

Due to incomplete restoration of pulmonary immunity with ART, HIV-infected individuals continue to have high burdens of pulmonary comorbidities, including chronic obstructive pulmonary disease (COPD) (34, 35), lung cancer (36–38), pulmonary fibrosis (39) and pulmonary emphysema (39–41). Overall, the complex respiratory tract microbiome, including the lung mycobiome, may play an important role in lung disease (42).

Previous studies have indicated that the diversity and composition of the lung microbiome in HIV-infected patients are altered compared with HIV-uninfected individuals (43–45) (**Table 1**). In addition, studies have also reported alterations in the respiratory tract mycobiome in HIV-infected individuals. Bittinger et al. analyzed bronchoalveolar lavage (BAL) samples from 42 lung transplant patients, 19 HIV-positive patients, 13 patients with various pulmonary diseases and 12 healthy controls; only low levels of fungal reads were detected in the healthy individuals, and the fungi detected comprised taxa with little clinical significance, except for *Aspergillus*. Conversely, clinical pathogens such as *Pneumocystis*, *Cryptococcus*, and *Aspergillus* were found in BAL of HIV-infected subjects (30). Another study published by Cui et al. compared fungal communities in the respiratory tract from 24 healthy subjects and 32 HIV-infected subjects: 9 species were overrepresented in the BAL of HIV-infected subjects, including *Pneumocystis jirovecii*, *Junghuhnia nitida*, *Phlebia tremellosa*, *Oxyporus latemarginatus*, *Sebacina incrustans*, *Ceriporia lacerata*, *Pezizella discrete*, *Trametes hirsute*, and *Daedaleopsis confragosa (* 29). Of these species, *Pneumocystis jirovecii* and *Ceriporia lacerata* are known to be pulmonary pathogens associated with immunosuppression. In addition, *Pneumocystis jirovecii* pneumonia (PCP) is one of the most common opportunistic infections in PLWH. These data reveal that alterations in the respiratory tract mycobiome might be an important driver of opportunistic infection in HIV-infected individuals.

### The gut mycobiome in HIV infection

The gut microbiome is being progressively recognized as playing an important role in promoting immune activation and inflammation in HIV infection. Dysbiosis of the gut microbiome has been demonstrated in many studies (9, 46, 47), and such alterations of the gut microbiome composition in HIV infection might be attributed to the loss of appropriate innate and adaptive immune responses (48).

**Figure 1:**
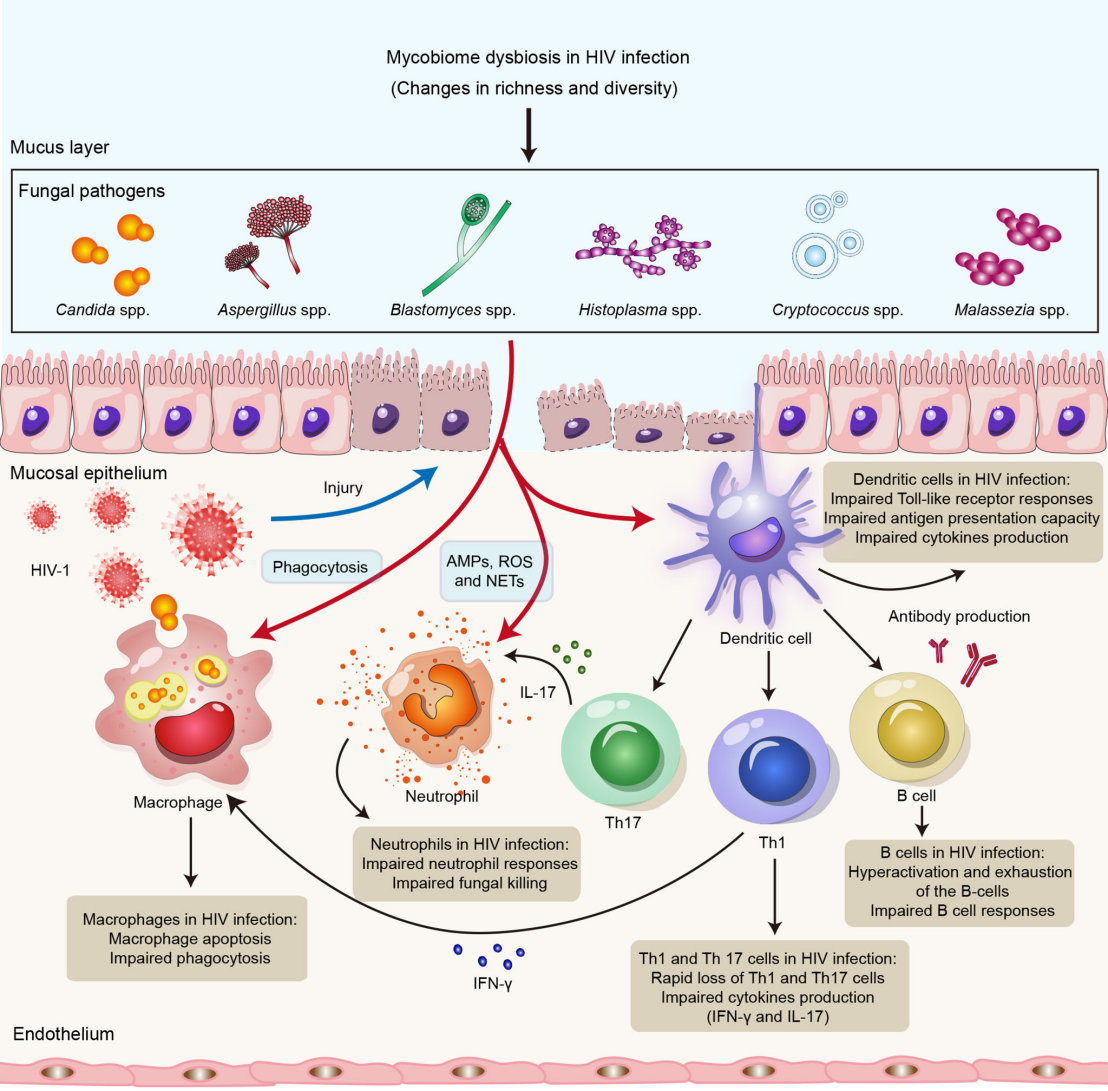
Impact of HIV infection on innate and adaptive immune responses to opportunistic fungal pathogens. HIV infection might lead to impairment of the innate and adaptive arms of the immune system, resulting in susceptibility to opportunistic fungal infections. Neutrophils control fungal infections through multiple mechanisms, including AMPs, ROS and NETs. Macrophages directly kill invading fungi through phagocytosis. DCs recognize fungal antigens by PRRs and also promote Th1 and Th17 immunity, as well as antibody production, to clear fungal infections. HIV can not only lead to impairment of neutrophil responses, phagocytosis of macrophages and the antigen presentation capacity of DCs but also cause defects in Th1, Th12 and B-cell responses. Abbreviations: AMPs, antimicrobial peptides; ROS, reactive oxygen species; NETs, neutrophil extracellular traps; DCs, dendritic cells; Th1, T helper 1 cell; Th17, T helper 1 cell; PRR, pattern recognition receptor.

In the human gut, the diversity of the fungal community is much lower than that of the bacterial microbiota (49). Fungi in the gastrointestinal tract are often ignored, as fungi comprise a tiny fraction of the gut microbes and most are unculturable. Previous studies have been shown that *Candida*, *Saccharomyces*, *Aspergillus*, *Cryptococcus*, *Malassezia*, *Cladosporium*, *Galactomyces* and *Trichosporon* can grow at 37°C and therefore have the potential to permanently colonize in the gut (50). In addition, although *Histoplasma* spp., *Coccidioides* spp. and *Blastomyces* spp. cannot colonize the mucosal surfaces, they can cause severe lung infections (51) (**Figure 1**). A recent study investigated the gut mycobiome of the Human Microbiome Project (HMP) cohort and revealed *Saccharomyces*, *Malassezia*, and *Candida* to be the most abundant genera present in this cohort (49). In a study of 96 healthy individuals, the most common genera in fecal samples were *Saccharomyces*, *Candida* and *Cladosporium* (present in 89%, 57% and 42%, respectively) (52). Another study showed that the most prevalent genus in healthy individuals is *Penicillium* (present in 73% of samples), followed by *Candida* and *Saccharomyces* (55% for both), *Mucor* (38%) and *Aspergillus* (35%) (53). Additionally, gut mycobiome alterations in HIV infection have been reported (**Table 1**). Gouba et al. found decreased fungal species diversity in HIV-infected individuals (54), showing significantly more abundance of *Candida* spp. in HIV-infected patients than in healthy individuals. *Candida albicans* in the gut can affect many processes, such as digestion and immunity (55). *Candida* spp. are more prevalent in HIV-infected individuals with diarrhea and recent antibiotic treatment than in healthy controls (54). Yin et al. showed that *Aspergillus* was the most abundant genus (49.92%) in the HIV-infected group, while the most abundant fungal genus was *Candida* (38.31%) in the healthy controls (33). Wu et al. found that 4 taxa from Ascomycota and 16 taxa from Basidiomycota were differentiated between HIV-infected individuals and HIV-uninfected controls in the fungal linear discriminant analysis (LDA) analysis. *Nectriaceae*, *Hypocreales*, and *Sordariomycetes* were the top 3 fungal taxa in HIV-infected individuals. While *Basidiomycota*, *Phallaceae*, and *Phallales* were particularly enriched in HIV-uninfected controls (32). Another study also compared the fungal populations of fecal samples from HIV-infected individuals and healthy controls; *Ascomycota*, *Pichia*, *Penicillium brevicompactum* and *Penicillium* were more abundant in healthy controls, whereas the abundances of *Candida albicans* and *Candida tropicalis* were enriched in HIV-infected individuals (31). In addition, Yin et al. demonstrated the relationship between CD4^+^ T-cell counts and the gut mycobiome in the HIV-infected participants (33). They found that patients with low CD4^+^ T-cell counts and patients with high CD4^+^ T-cell counts have different fungal community characteristics. *Eurotiomycetes* was significantly decreased and *Saccharomycetes* was significantly increased in the low CD4^+^ T-cell group compared to the high CD4^+^ T-cell group. At the genus level, *Candida* was significantly increased in the low CD4^+^ T-cell group, indicating a high risk of opportunistic infection. Moreover, unclassified *Aspergillaceae* and *Dirkmeia* were enriched in the high CD4^+^ T-cell group, while *Sordariales*, *Saccharomycetaceae*, and *Neocosmospora* were enriched in the low CD4^+^ T-cell group. It has been shown that members of the genus *Neocosmospora* can lead to lung infections in liver transplant patients (56) and contain highly prevalent and aggressive fungal pathogens (57), suggesting that the immune T-cell reduction might expose patients to a state of high-risk infection.

## Common fungal diseases in HIV infection

### Oropharyngeal candidiasis

OPC is the most common opportunistic fungal infection in the early stages of HIV infection (58–60). OPC is caused by various *Candida* species, with *Candida albicans* being the most prevalent species isolated from HIV-infected patients (58, 59, 61, 62). In patients with a new diagnosis of HIV, the prevalence of OPC is reportedly 27% (63). It has also been found that OPC occurs in approximately 80%-90% of HIV-infected individuals in different phases of the disease (64). Although the occurrence of OPC in HIV infection has significantly decreased after the introduction of ART, it remains a common opportunistic infection in HIV (65, 66).

The incidence of OPC in HIV infection is influenced by a multitude of factors (67), including immune status (68), bacteriome–mycobiome interaction (69), anti-fungal therapy and ART (70). Several studies have shown that a lower CD4^+^ T-cell count, especially below 200 cells/μl, is strongly associated with increased occurrence of OPCs (71–74). A reduction in CD4^+^ T cells, especially IL-17-producing cells, in the oral mucosa may be associated with susceptibility to OPC (75). In addition, impairment of oral immunity by the reduction of salivary components such as salivary IgA, defensins, and some cytokines might lead to the onset of OPC (76). However, host defense mechanisms executed by keratinocytes, calprotectin, CD8^+^ T cells, and phagocytes partly compensate for these defects, which may play a key role in controlling *C. albicans* proliferation and preventing systemic dissemination in HIV infection (68).

### *Pneumocystis jirovecii* pneumonia

PCP is one of the most common opportunistic fungal infections in immunocompromised individuals and HIV-infected individuals (77, 78). In the late 1980s, PCP occurred in approximately 75% of HIV-infected individuals (79), though the incidence of HIV-associated PCP has decreased dramatically with the implementation of ART and chemoprophylaxis (80). Nonetheless, PCP continues to be a serious problem in HIV-infected patients who are undiagnosed and untreated or in those with ART failure (81). It is speculated that there are more than 400,000 cases of PCP worldwide each year (82, 83).

As mentioned above, PCP tends to occur most frequently when the CD4^+^ T-cell count is below 200 cells/μl (84–86), and CD4^+^ T cells, CD8^+^ T cells, neutrophils, alveolar macrophages and soluble mediators have been implicated in clearance of PCP (87). Carmona et al. demonstrated that *Pneumocystis*-derived β-glucans activate dendritic cells (DCs) through the Fas ligand (FasL) mechanism and the Dectin-1 receptor, leading to increased expression of costimulatory molecules and T helper 1 (Th1) cell activation (88). Another study found that DCs stimulated by cell-surface β-glucan components of *Pneumocystis* interact with lymphocytes to produce IL-17 and IL-22 (89). Th1, Th2 and Th17 responses are essential in *Pneumocystis* clearance and contribute to host protection against this pathogen (90). However, Th2 and Th17 responses also play a role in *Pneumocystis*-driven pathology (90). In addition, the cytokines produced by CD4^+^ T cells, such as IFN-γ, also are important for the control of PCP (87, 90).

### Cryptococcal meningitis

Cryptococcal meningitis (CM) is one of the most common opportunistic infections in the late stage of AIDS (91), and *Cryptococcus neoformans* is the most common cause of death in HIV-infected individuals. An estimated 223,100 cases of CM occur globally each year, resulting in 181,100 deaths; 135,900 occur in sub-Saharan Africa, and CM accounts for 15% of all AIDS-related deaths (91). Despite effective ART and antifungal drugs, the mortality rate of CM in AIDS patients is still as high as 30%-50%, especially in patients in resource-poor areas (92–94).

Evidence suggests that host immune responses to cryptococcosis play a critical role in disease progression (95–97). CM can occur following primary lung infection or by reactivation and dissemination of latent pulmonary infection in the setting of cell-mediated immunodeficiency when CD4^+^ T-cell counts are <100 cells/μl in the late-stages of HIV-infection (98). Previous studies have shown that CD4^+^ T cells possibly mediate protective host immunity against cryptococcal *via* production of Th1-type cytokine responses, including IL-2, IL-12, tumor necrosis factor alpha (TNF-α), and IFN-γ, which play an essential role in recruitment of lymphocytes and phagocytes to clear the infection (99, 100). Higher levels of IFN-γ in cerebrospinal fluid (CSF) are associated with a faster rate of fungal clearance and lower fungal burdens (101, 102). Moreover, higher pre-ART levels of IL-4 and IL-17 and lower TNF-α, granulocyte colony-stimulating factor (G-CSF), granulocyte-macrophage colony-stimulating factor (GM-CSF) and vascular endothelial growth factor (VEGF) might predict future immune reconstitution inflammatory syndrome (IRIS) (103).

### *Talaromyces marneffei* infection

Talaromycosis is an invasive fungal disease caused by the opportunistic fungus *Talaromyces marneffei* (TM) and is prevalent mainly in Southeast Asia. Since the HIV pandemic, the prevalence of talaromycosis has rapidly increased, especially in areas of Southeast Asia, including Thailand, Vietnam and Myanmar, and East Asia, including South China, Hong Kong, Taiwan, and northeastern India (104). A recent study showed that the prevalence of TM infection in Asia was 3.6% in HIV-infected individuals (105). The prevalence of TM infection in HIV-infected individuals has been reported to be 6.4% in Vietnam, 3.9% in Thailand, 3.3% in China, 3.2% in India, and 2.1% in Malaysia (105). Furthermore, mortality rate of TM infection is reportedly higher than that of most HIV-related complications in PLWH (106). Although ART has led to a decline in the incidence of TM infection, it remains a major problem in undiagnosed and untreated HIV-infected individuals (81).

Innate and acquired immune responses play a crucial role in controlling TM infection (107). Innate immune cells, including monocytes (108), macrophages (109, 110), polymorphonuclear neutrophils (PMNs) (111), and DCs (112) have been shown to play an essential role in combating TM. These innate immune cells promote clearance of TM infection by producing proinflammatory cytokines such as IL-1β, TNF-α, and IFN-γ and anti-inflammatory cytokines such as IL-10 (107). A recent study also demonstrated that single-nucleotide polymorphisms (SNPs) in TLR2 might contribute to increased susceptibility and severity of TM in Han Chinese populations (113). Moreover, another study found that severe TM infection in Southeast Asia may be related to the high prevalence of anti-IFN-γ autoantibody-associated HLA-DRB1*16:02 and HLA-DQB1*05:02 alleles (114). A previous study found that HIV-infected individuals with CD4^+^ T-cell counts below 200 cells/μl had a higher risk of TM infection (105). In general, immune deficiencies that reduce CD4^+^ T cells and IFN-γ, IL-12, and IL-17 functions may be predisposing factors for TM infection, as evidenced by the high infection burden in advanced HIV-infected individuals, highlighting the important roles of Th1 and Th17 responses in host resistance to TM infection (115).

### Histoplasmosis

Histoplasmosis is caused by *Histoplasma capsulatum*, which is a common endemic mycosis in PLWH (116). Globally, epidemic distribution of histoplasmosis mainly in regions of the Americas (81). In addition, histoplasmosis is also endemic in many Asia areas, including Southeast Asia, India, and China along the Yangtze River (117, 118). With the spread of HIV, the case-fatality rates of disseminated histoplasmosis increased among culture-positive cases, ranging from 10% to 53% (119). Disseminated histoplasmosis has been neglected due to its nonspecific symptoms, frequent misdiagnosis as tuberculosis, and insensitive diagnostic methods (120).

HIV-infected individuals are at greatly increased risk of developing histoplasmosis, especially those with CD4^+^ T-cell counts <200 cells/μl (121). *Histoplasma capsulatum* yeasts can infect macrophages and survive within phagocytic cells (122). The strategies of *Histoplasma capsulatum* against macrophages might include immune response evasion on entry, inactivation of nitrogen and oxygen reactive species, hindrance of lysosomal pH reduction, production of siderophore, prevention of phagolysosomal fusion, and induction of apoptosis (123). In addition to the control of *Histoplasma capsulatum* infection by cellular immune response, the roles of antibodies in the serodiagnosis of histoplasmosis have also been proposed. Almeida et al. characterized *Histoplasma capsulatum* proteins specifically recognized by antibodies in serum samples from histoplasmosis patients by an immunoproteomic approach (123).

### Aspergillosis

Aspergillosis is a life-threatening fungal disease in immunocompromised individuals, including PLWH. Previous study has been shown that the incidence of aspergillosis was 3.5 cases per 1000 person-years among 35,252 HIV-infected individuals (124). Although aspergillosis occurs uncommonly in HIV-infected individuals, it is associated with a short lifespan after diagnosis.

It has been shown that older people, people with severe immunosuppression or advanced HIV disease, and people with leukopenia and neutropenia are at increased risk of developing aspergillosis (124). Before the advent of ART, invasive aspergillosis in HIV-infected individuals tended to occur when CD4 T-cell counts <100 cells/µl, especially in patients with prior or concomitant opportunistic infections (125). Therefore, modulation of host immunity plays a critical role in the control of aspergillosis. Animal studies in aspergillosis have also demonstrated beneficial effects of G-CSF, GM-CSF, IFN-γ, and monoclonal antibodies (126).

## Antifungal immunity in HIV infection

### Innate antifungal immunity in HIV infection

The innate immune response is the first line of defense against fungal infections (127). Innate immune cells, such as neutrophils, monocytes, macrophages, and dendritic cells, are known to have a crucial function in recognizing and clearing fungi, inducing protective immune responses, and initiating adaptive immune responses during fungal infections (127, 128). Neutrophils control fungal infections through multiple mechanisms, including production of granule proteins, antimicrobial peptides (AMPs) and reactive oxygen species (ROS) and formation of neutrophil extracellular traps (129, 130). Indeed, neutrophils are critical cells against *Candida* spp. and *Aspergillus* spp (131). Macrophages not only directly kill invading fungi through phagocytosis but also initiate and regulate downstream immune responses to clear fungal infections by releasing cytokines, presenting antigens, and recruiting other immune cells (132). Fungal antigens are also recognized by DCs mediated by CLRs, including dectin-1, dectin-2 and DC-SIGN, as well as TLRs, including TLR2, TLR4 and TLR9 (133). DCs might also collaborate with other immune cells, such as Group 2 innate lymphoid cells (ILC2s), to promote innate antifungal immune responses and regulate adaptive immune responses (130). Nevertheless, the effect of HIV infection on innate antifungal immunity remains unclear.

HIV does not directly infect neutrophils but can cause impaired neutrophil responses, leading to impaired bacterial and fungal killing, which might result in increased susceptibility to bacterial infections and mycoses (134). Enomoto et al. found that anti-cryptococcal activity in HIV-infected patients is enhanced by administration of granulocyte colony stimulating factor (G-CSF) to enhance neutrophil defense (135). Moreover, Kalem et al. showed that HIV-1 infection of THP-1 macrophages increases the rate of *Cryptococcus neoformans* cell phagocytosis (136); these authors revealed that macrophages infected with HIV-1 alone might upregulate production of TNF-α and activate NF-κB signaling but that *Cryptococcus neoformans* coinfection rapidly represses this proinflammatory response (136). In addition, HIV-infected macrophages might contribute to increased susceptibility to opportunistic fungal infections (137). Studies have indicated that HIV infection of macrophages impairs the phagocytosis and killing of *Pneumocystis jirovecii (* 138), *Candida albicans (* 139) and *Aspergillus fumigatus (* 140). A possible reason is that the HIV-1 accessory proteins Nef and Tat downregulate the mannose receptor expressed on the surface of macrophages. It has also been shown that HIV-1 reduces the number of DCs and disrupt their function. In HIV infection, DCs have a reduced ability to present antigens and stimulate T-cell proliferation and show a partially activated phenotype and impaired TLR responses (141, 142). T-cell proliferation in HIV-infected individuals might be inhibited by plasmacytoid DCs (pDCs) *via* induction of indoleamine-2,3-dioxygenase (IDO) (143). IDO expression by pDCs also blocks T-cell differentiation into Th17 cells, which might have a negative effect in adaptive antifungal immunity and predispose patients toward opportunistic infections, such as fungal infections with *C. albicans* and *C. neoformans (* 141). Overall, HIV infection might lead to quantitative and qualitative deficiencies in innate antifungal immunity (**Figure 1**).

### Adaptive antifungal immunity in HIV infection

CD4^+^ T cells are generally considered to play an important role in defense against fungal infections. The importance of Th1 and Th17 responses in antifungal defense mechanisms has been described (144). It is well known that the Th1 response provides protective immunity mainly through production of proinflammatory cytokines, such as IFN-γ, IL-2, IL-12, and TNF-α (144). The IFN-γ produced by Th1 cells activates phagocytes, such as macrophages, and promotes phagocytosis, MHC-II molecule upregulation and antigen presentation by APCs (145). Enhanced protection against *Aspergillosis*, *Cryptococcosis*, and coccidioidomycosis has been demonstrated in patients receiving IFN-γ immunotherapy (146). Th17 cells produce cytokines, including IL-17A, IL-17F, and IL-22, which promote neutrophil recruitment and fungicidal activity and induce production of AMPs from epithelial cells and keratinocytes to prevent fungal overgrowth (145). The Th17 response has been shown to play an essential role in promoting clearance of fungi, such as *Candida albicans* and *Malassezia* spp (147). In addition, antibodies are important in limiting the fungal burden and its clearance (148). Antibodies can defend against fungal pathogens through direct mechanisms, including inhibition of fungal pathogen growth or fungicidal activity, and indirect mechanisms, including opsonization, complement pathway activation and antibody-directed cell toxicity (ADCC) (146). Antibody responses to *Cryptococcus neoformans (* 149) and *Candida albicans (* 150) have been reported.

HIV infection leads to a rapid and massive reduction in CD4^+^ T cells. One recent study showed that levels of Th1 cytokines in CSF, including IL-12 and TNF-α, correlate positively with HIV-associated cryptococcal meningitis (151). Moreover, in HIV-infected individuals, IFN-γ produced by Th1 cells plays an important function in improving the antifungal immune response to cryptococcal infection (102) and oral candidiasis (152). Jarvis et al. found that the Th1 responses of *Cryptococcus*-specific CD4^+^ T cells play a key role in promoting circulating lymphocyte and monocyte recruitment to the central nervous system (CNS), CNS macrophage and microglial activation and organism clearance (153). In addition to the Th1 response, Th17 cells are critical in defense against bacterial and fungal infections at mucosal sites (154, 155). However, Liu et al. found that Th17-associated functions (IL-22, IL-17 and IL-2) of *Candida albicans*-specific CD4 T cells are disrupted in early HIV infection (156). Early massive loss of Th17 cells in HIV infection has also been shown to be a likely cause of the high prevalence of chronic mucocutaneous candidiasis in people with early HIV infection (157). Therefore, mucosal candidiasis susceptibility in HIV infection may be attributed to Th17-cell depletion.

HIV-1 replication might lead to abnormalities in all major lymphocyte populations as well as hyperactivation and exhaustion of the B-cell compartment (158). Studies have found that impaired B-cell responses due to HIV infection might affect B-cell responses in cryptococcal coinfection (159, 160). Moreover, levels of antibodies, such as plasma IgM, laminarin (Lam)-binding IgM and IgG, are significantly lower in HIV-infected individuals who develop *Cryptococcus*-associated IRIS than in those who do not, supporting the role for antibody immunity in cryptococcosis (161). Immune status is also important in antibody responses to *Pneumocystis jirovecii (* 162). A previous study showed that IgM antibody responses to *Pneumocystis jirovecii* major surface glycoprotein (Msg), including MsgC1 (carboxyl terminus), MsgC3, MsgC8 and MsgC9, were significantly lower in HIV-infected individuals than in HIV-uninfected controls (163). Taken together, these findings suggest that competent adaptive immune responses are crucial for defense against fungal infections and that HIV infection might lead to impaired antifungal immunity (**Figure 1**).

## Conclusion

Our review discusses recent findings on alterations in the mycobiome in the setting of HIV infection. The mycobiome contributes greatly to opportunistic infections in individuals with advanced HIV infection. Despite widespread use of ART, fungal opportunistic infections are the leading cause of HIV-related death globally. It is evident that human immune responses play a critical role in defense against fungal infection. We review the impact of HIV infection on host innate and adaptive antifungal immunity, contributing to a better understanding of the underlying immunopathogenesis of fungal infections in HIV infection. In addition, further efforts to develop new diagnostics and global access to antifungal drugs and other effective therapies are needed to enable early diagnosis and treatment of fungal infections.

## Author contributions

BS and TZ conceptualized and supervised the whole study, SL, XY, HW, BS, and TZ searched the literature, contributed to the analysis and provided important scientific input. SL, CM and BS wrote the first draft and revised version of the manuscript. All authors contributed to the article and approved the submitted version.

## Funding

This project is financially supported by the National Natural Science Foundation of China (NSFC, 82072271 and 81974303), Beijing Natural Science Foundation (Z220018), the High-Level Public Health Specialized Talents Project of Beijing Municipal Health Commission (2022-1-007 and 2022-2-018), the “Climbing the peak (Dengfeng)” Talent Training Program of Beijing Hospitals Authority (DFL20191701), the Beijing Health Technologies Promotion Program (BHTPP2020), and Beijing Key Laboratory for HIV/AIDS Research (BZ0089). The funders had no role in study design, data collection and analysis, decision to publish, or preparation of the manuscript.

## Conflict of interest

The authors declare that the research was conducted in the absence of any commercial or financial relationships that could be construed as a potential conflict of interest.

## Publisher’s note

All claims expressed in this article are solely those of the authors and do not necessarily represent those of their affiliated organizations, or those of the publisher, the editors and the reviewers. Any product that may be evaluated in this article, or claim that may be made by its manufacturer, is not guaranteed or endorsed by the publisher.

## References

[1] Brown GD Denning DW Levitz SM. Tackling human fungal infections. Science (2012) 336(6082):647. doi: 10.1126/science.1222236 22582229

[2] Stop neglecting fungi. Nat Microbiol (2017) 2:17120. doi: 10.1038/nmicrobiol.2017.120 28741610

[3] Tirado-Sanchez A Gonzalez GM Bonifaz A. Endemic mycoses: epidemiology and diagnostic strategies. Expert Rev Anti Infect Ther (2020) 18(11):1105–17. doi: 10.1080/14787210.2020.1792774 32620065

[4] Li S Zhu J Su B Wei H Chen F Liu H. Alteration in oral microbiome among men who have sex with men with acute and chronic HIV infection on antiretroviral therapy. Front Cell Infect Microbiol (2021) 11:695515. doi: 10.3389/fcimb.2021.695515 34336719 PMC8317457

[5] Li S Su B He QS Wu H Zhang T. Alterations in the oral microbiome in HIV infection: causes, effects and potential interventions. Chin Med J (Engl) (2021) 134(23):2788–98. doi: 10.1097/CM9.0000000000001825 PMC8667981 34670249

[6] Zhou JJ Zhai J Zhou H Chen Y Guerra S Robey I. Supraglottic lung microbiome taxa are associated with pulmonary abnormalities in an HIV longitudinal cohort. Am J Respir Crit Care Med (2020) 202(12):1727–31. doi: 10.1164/rccm.202004-1086LE PMC7737582 32783620

[7] Cribbs SK Crothers K Morris A. Pathogenesis of HIV-related lung disease: Immunity, infection, and inflammation. Physiol Rev (2020) 100(2):603–32. doi: 10.1152/physrev.00039.2018 31600121

[8] Nganou-Makamdop K Talla A Sharma AA Darko S Ransier A Laboune F. Translocated microbiome composition determines immunological outcome in treated HIV infection. Cell (2021) 184(15):3899–3914.e3816. doi: 10.1016/j.cell.2021.05.023 34237254 PMC8316372

[9] Rocafort M Noguera-Julian M Rivera J Pastor L Guillen Y Langhorst J. Evolution of the gut microbiome following acute HIV-1 infection. Microbiome (2019) 7(1):73. doi: 10.1186/s40168-019-0687-5 31078141 PMC6511141

[10] Armstrong-James D Meintjes G Brown GD. A neglected epidemic: fungal infections in HIV/AIDS. Trends Microbiol (2014) 22(3):120–7. doi: 10.1016/j.tim.2014.01.001 24530175

[11] Armstrong-James D Bicanic T Brown GD Hoving JC Meintjes G Nielsen K. AIDS-related mycoses: Current progress in the field and future priorities. Trends Microbiol (2017) 25(6):428–30. doi: 10.1016/j.tim.2017.02.013 PMC7549875 28454846

[12] Hoving JC Brown GD Gomez BL Govender NP Limper AH May RC. AIDS-related mycoses: Updated progress and future priorities. Trends Microbiol (2020) 28(6):425–8. doi: 10.1016/j.tim.2020.01.009 32396822

[13] Garcia-Carnero LC Perez-Garcia LA Martinez-Alvarez JA Reyes-Martinez JE Mora-Montes HM. Current trends to control fungal pathogens: exploiting our knowledge in the host-pathogen interaction. Infect Drug Resist (2018) 11:903–13. doi: 10.2147/IDR.S170337 PMC6037146 30013373

[14] Casadevall A. Immunity to invasive fungal diseases. Annu Rev Immunol (2022) 40:121–41. doi: 10.1146/annurev-immunol-101220-034306 35007128

[15] Hoenigl M. Fungal translocation: A driving force behind the occurrence of non-AIDS events? Clin Infect Dis (2020) 70(2):242–4. doi: 10.1093/cid/ciz215 PMC6938977 30861074

[16] Ramendra R Isnard S Mehraj V Chen J Zhang Y Finkelman M. Circulating LPS and (1–>3)-beta-D-Glucan: A folie a deux contributing to HIV-associated immune activation. Front Immunol (2019) 10:465. doi: 10.3389/fimmu.2019.00465 30967860 PMC6430738

[17] Cui L Morris A Ghedin E. The human mycobiome in health and disease. Genome Med (2013) 5(7):63. doi: 10.1186/gm467 23899327 PMC3978422

[18] Rolling T Hohl TM Zhai B. Minority report: the intestinal mycobiota in systemic infections. Curr Opin Microbiol (2020) 56:1–6. doi: 10.1016/j.mib.2020.05.004 32599521 PMC7744423

[19] Gu Y Zhou G Qin X Huang S Wang B Cao H. The potential role of gut mycobiome in irritable bowel syndrome. Front Microbiol (2019) 10:1894. doi: 10.3389/fmicb.2019.01894 31497000 PMC6712173

[20] Guo Y Xia W Wei F Feng W Duan J Sun X. Salivary microbial diversity at different stages of human immunodeficiency virus infection. Microb Pathog (2021) 155:104913. doi: 10.1016/j.micpath.2021.104913 33915204

[21] Li J Chang S Guo H Ji Y Jiang H Ruan L. Altered salivary microbiome in the early stage of HIV infections among young Chinese men who have sex with men (MSM). Pathogens (2020) 9(11):960. doi: 10.3390/pathogens9110960 33228000 PMC7699166

[22] Guo Y Huang X Sun X Yu Y Wang Y Zhang B. The underrated salivary virome of men who have sex with men infected with HIV. Front Immunol (2021) 12:759253. doi: 10.3389/fimmu.2021.759253 34925329 PMC8674211

[23] Sodre CS Rodrigues PMG Vieira MS Marques Paes da Silva A Goncalves LS Ribeiro MG. Oral mycobiome identification in atopic dermatitis, leukemia, and HIV patients - a systematic review. J Oral Microbiol (2020) 12(1):1807179. doi: 10.1080/20002297.2020.1807179 32944157 PMC7482892

[24] Ghannoum MA Jurevic RJ Mukherjee PK Cui F Sikaroodi M Naqvi A. Characterization of the oral fungal microbiome (mycobiome) in healthy individuals. PLos Pathog (2010) 6(1):e1000713. doi: 10.1371/journal.ppat.1000713 20072605 PMC2795202

[25] Mukherjee PK Chandra J Retuerto M Sikaroodi M Brown RE Jurevic R. Oral mycobiome analysis of HIV-infected patients: identification of pichia as an antagonist of opportunistic fungi. PLos Pathog (2014) 10(3):e1003996. doi: 10.1371/journal.ppat.1003996 24626467 PMC3953492

[26] Hager CL Ghannoum MA. The mycobiome in HIV. Curr Opin HIV AIDS (2018) 13(1):69–72. doi: 10.1097/COH.0000000000000432 29028668 PMC5805152

[27] Chang S Guo H Li J Ji Y Jiang H Ruan L. Comparative analysis of salivary mycobiome diversity in human immunodeficiency virus-infected patients. Front Cell Infect Microbiol (2021) 11:781246. doi: 10.3389/fcimb.2021.781246 34926323 PMC8671614

[28] Fidel PL Jr. Thompson ZA Lilly EA Granada C Treas K Dubois KR 3rd. Effect of HIV/HAART and other clinical variables on the oral mycobiome using multivariate analyses. mBio (2021) 12(2):e00294-21. doi: 10.1128/mBio.00294-21 33758093 PMC8092233

[29] Cui L Lucht L Tipton L Rogers MB Fitch A Kessinger C. Topographic diversity of the respiratory tract mycobiome and alteration in HIV and lung disease. Am J Respir Crit Care Med (2015) 191(8):932–42. doi: 10.1164/rccm.201409-1583OC PMC4435454 25603113

[30] Bittinger K Charlson ES Loy E Shirley DJ Haas AR Laughlin A. Improved characterization of medically relevant fungi in the human respiratory tract using next-generation sequencing. Genome Biol (2014) 15(10):487. doi: 10.1186/s13059-014-0487-y 25344286 PMC4232682

[31] Hamad I Abou Abdallah R Ravaux I Mokhtari S Tissot-Dupont H Michelle C. Metabarcoding analysis of eukaryotic microbiota in the gut of HIV-infected patients. PLos One (2018) 13(1):e0191913. doi: 10.1371/journal.pone.0191913 29385188 PMC5791994

[32] Wu X Xu Y Li Q Yang F He S Li Y. Gut dysbiosis of bacteria and fungi associated with human immunodeficiency virus infection. Chin Med J (Engl) (2022) 135(11):1376–8. doi: 10.1097/CM9.0000000000002194 PMC9433070 35830264

[33] Yin Y Tuohutaerbieke M Feng C Li X Zhang Y Xu Q. Characterization of the intestinal fungal microbiome in HIV and HCV mono-infected or Co-infected patients. Viruses (2022) 14(8):1811. doi: 10.3390/v14081811 36016433 PMC9412373

[34] Lalloo UG Pillay S Mngqibisa R Abdool-Gaffar S Ambaram A. HIV And COPD: a conspiracy of risk factors. Respirology (2016) 21(7):1166–72. doi: 10.1111/resp.12806 27237114

[35] Bigna JJ Kenne AM Asangbeh SL Sibetcheu AT. Prevalence of chronic obstructive pulmonary disease in the global population with HIV: a systematic review and meta-analysis. Lancet Glob Health (2018) 6(2):e193–202. doi: 10.1016/S2214-109X(17)30451-5 29254748

[36] Sigel K Wisnivesky J Gordon K Dubrow R Justice A Brown ST. HIV As an independent risk factor for incident lung cancer. AIDS (2012) 26(8):1017–25. doi: 10.1097/QAD.0b013e328352d1ad PMC3580210 22382152

[37] Sigel K Makinson A Thaler J. Lung cancer in persons with HIV. Curr Opin HIV AIDS (2017) 12(1):31–8. doi: 10.1097/COH.0000000000000326 PMC5241551 27607596

[38] Sigel K Wisnivesky J Crothers K Gordon K Brown ST Rimland D. Immunological and infectious risk factors for lung cancer in US veterans with HIV: a longitudinal cohort study. Lancet HIV (2017) 4(2):e67–73. doi: 10.1016/S2352-3018(16)30215-6 PMC5444465 27916584

[39] Leader JK Crothers K Huang L King MA Morris A Thompson BW. Risk factors associated with quantitative evidence of lung emphysema and fibrosis in an HIV-infected cohort. J Acquir Immune Defic Syndr (2016) 71(4):420–7. doi: 10.1097/QAI.0000000000000894 PMC4770858 26914911

[40] Attia EF Akgun KM Wongtrakool C Goetz MB Rodriguez-Barradas MC Rimland D. Increased risk of radiographic emphysema in HIV is associated with elevated soluble CD14 and nadir CD4. Chest (2014) 146(6):1543–53. doi: 10.1378/chest.14-0543 PMC4251616 25080158

[41] Petrache I Diab K Knox KS Twigg HL 3rd Stephens RS Flores S. HIV Associated pulmonary emphysema: a review of the literature and inquiry into its mechanism. Thorax (2008) 63(5):463–9. doi: 10.1136/thx.2007.079111 PMC13200782 18443163

[42] Seed PC. The human mycobiome. Cold Spring Harb Perspect Med (2014) 5(5):a019810. doi: 10.1101/cshperspect.a019810 25384764 PMC4448585

[43] Twigg HL 3rd Knox KS Zhou J Crothers KA Nelson DE Toh E. Effect of advanced HIV infection on the respiratory microbiome. Am J Respir Crit Care Med (2016) 194(2):226–35. doi: 10.1164/rccm.201509-1875OC PMC5003215 26835554

[44] Twigg HL 3rd Weinstock GM Knox KS. Lung microbiome in human immunodeficiency virus infection. Transl Res (2017) 179:97–107. doi: 10.1016/j.trsl.2016.07.008 27496318 PMC5164960

[45] Alexandrova Y Costiniuk CT Jenabian MA. Pulmonary immune dysregulation and viral persistence during HIV infection. Front Immunol (2021) 12:808722. doi: 10.3389/fimmu.2021.808722 35058937 PMC8764194

[46] Dillon SM Lee EJ Kotter CV Austin GL Dong Z Hecht DK. An altered intestinal mucosal microbiome in HIV-1 infection is associated with mucosal and systemic immune activation and endotoxemia. Mucosal Immunol (2014) 7(4):983–94. doi: 10.1038/mi.2013.116 PMC4062575 24399150

[47] Armstrong AJS Shaffer M Nusbacher NM Griesmer C Fiorillo S Schneider JM. An exploration of prevotella-rich microbiomes in HIV and men who have sex with men. Microbiome (2018) 6(1):198. doi: 10.1186/s40168-018-0580-7 30396369 PMC6219090

[48] Crakes KR Jiang G. Gut microbiome alterations during HIV/SIV infection: Implications for HIV cure. Front Microbiol (2019) 10:1104. doi: 10.3389/fmicb.2019.01104 31191468 PMC6539195

[49] Nash AK Auchtung TA Wong MC Smith DP Gesell JR Ross MC. The gut mycobiome of the human microbiome project healthy cohort. Microbiome (2017) 5(1):153. doi: 10.1186/s40168-017-0373-4 29178920 PMC5702186

[50] Iliev ID Leonardi I. Fungal dysbiosis: immunity and interactions at mucosal barriers. Nat Rev Immunol (2017) 17(10):635–46. doi: 10.1038/nri.2017.55 PMC5724762 28604735

[51] Cutler JE Deepe GS Jr. Klein BS. Advances in combating fungal diseases: vaccines on the threshold. Nat Rev Microbiol (2007) 5(1):13–28. doi: 10.1038/nrmicro1537 17160002 PMC2214303

[52] Hoffmann C Dollive S Grunberg S Chen J Li H Wu GD. Archaea and fungi of the human gut microbiome: correlations with diet and bacterial residents. PLos One (2013) 8(6):e66019. doi: 10.1371/journal.pone.0066019 23799070 PMC3684604

[53] Mar Rodriguez M Perez D Javier Chaves F Esteve E Marin-Garcia P Xifra G. Obesity changes the human gut mycobiome. Sci Rep (2015) 5:14600. doi: 10.1038/srep14600 26455903 PMC4600977

[54] Gouba N Drancourt M. Digestive tract mycobiota: a source of infection. Med Mal Infect (2015) 45(1-2):9–16. doi: 10.1016/j.medmal.2015.01.007 25684583

[55] Musumeci S Coen M Leidi A Schrenzel J. The human gut mycobiome and the specific role of candida albicans: where do we stand, as clinicians? Clin Microbiol Infect (2022) 28(1):58–63. doi: 10.1016/j.cmi.2021.07.034 34363944

[56] Yamasmith E Chongtrakool P Chayakulkeeree M. Isolated pulmonary fusariosis caused by neocosmospora pseudensiformis in a liver transplant recipient: A case report and review of the literature. Transpl Infect Dis (2020) 22(6):e13344. doi: 10.1111/tid.13344 32479709

[57] Sandoval-Denis M Crous PW. Removing chaos from confusion: assigning names to common human and animal pathogens in neocosmospora. Persoonia (2018) 41:109–29. doi: 10.3767/persoonia.2018.41.06 PMC6344815 30728601

[58] Khedri S Santos ALS Roudbary M Hadighi R Falahati M Farahyar S. Iranian HIV/AIDS patients with oropharyngeal candidiasis: identification, prevalence and antifungal susceptibility of candida species. Lett Appl Microbiol (2018) 67(4):392–9. doi: 10.1111/lam.13052 30019443

[59] Hosain Pour A Salari S Ghasemi Nejad Almani P. Oropharyngeal candidiasis in HIV/AIDS patients and non-HIV subjects in the southeast of Iran. Curr Med Mycol (2018) 4(4):1–6. doi: 10.18502/cmm.4.4.379 PMC6386505 30815610

[60] Pienaar ED Young T Holmes H. Interventions for the prevention and management of oropharyngeal candidiasis associated with HIV infection in adults and children. Cochrane Database Syst Rev (2010) 11):CD003940. doi: 10.1002/14651858.CD003940.pub3 PMC7156835 21069679

[61] Kwamin F Nartey NO Codjoe FS Newman MJ. Distribution of candida species among HIV-positive patients with oropharyngeal candidiasis in Accra, Ghana. J Infect Dev Ctries (2013) 7(1):41–5. doi: 10.3855/jidc.2442 23324819

[62] Rafat Z Sasani E Salimi Y Hajimohammadi S Shenagari M Roostaei D. The prevalence, etiological agents, clinical features, treatment, and diagnosis of HIV-associated oral candidiasis in pediatrics across the world: A systematic review and meta-analysis. Front Pediatr (2021) 9:805527. doi: 10.3389/fped.2021.805527 35004551 PMC8740125

[63] Patel PK Erlandsen JE Kirkpatrick WR Berg DK Westbrook SD Louden C. The changing epidemiology of oropharyngeal candidiasis in patients with HIV/AIDS in the era of antiretroviral therapy. AIDS Res Treat (2012) 2012:262471. doi: 10.1155/2012/262471 22970352 PMC3434376

[64] Vazquez JA. Therapeutic options for the management of oropharyngeal and esophageal candidiasis in HIV/AIDS patients. HIV Clin Trials (2000) 1(1):47–59. doi: 10.1310/T7A7-1E63-2KA0-JKWD 11590489

[65] Thompson GR Patel PK Kirkpatrick WR Westbrook SD Berg D Erlandsen J. Oropharyngeal candidiasis in the era of antiretroviral therapy. Oral Surg Oral Med Oral Pathol Oral Radiol Endod (2010) 109(4):488–95. doi: 10.1016/j.tripleo.2009.11.026 PMC2843789 20156694

[66] Taverne-Ghadwal L Kuhns M Buhl T Schulze MH Mbaitolum WJ Kersch L. Epidemiology and prevalence of oral candidiasis in HIV patients from Chad in the post-HAART era. Front Microbiol (2022) 13:844069. doi: 10.3389/fmicb.2022.844069 35250957 PMC8891798

[67] Patil S Majumdar B Sarode SC Sarode GS Awan KH. Oropharyngeal candidosis in HIV-infected patients-an update. Front Microbiol (2018) 9:980. doi: 10.3389/fmicb.2018.00980 29867882 PMC5962761

[68] de Repentigny L Lewandowski D Jolicoeur P. Immunopathogenesis of oropharyngeal candidiasis in human immunodeficiency virus infection. Clin Microbiol Rev (2004) 17(4):729–59. doi: 10.1128/CMR.17.4.729-759.2004 PMC523562 15489345

[69] Oever JT Netea MG. The bacteriome-mycobiome interaction and antifungal host defense. Eur J Immunol (2014) 44(11):3182–91. doi: 10.1002/eji.201344405 25256886

[70] Patton LL. Current strategies for prevention of oral manifestations of human immunodeficiency virus. Oral Surg Oral Med Oral Pathol Oral Radiol (2016) 121(1):29–38. doi: 10.1016/j.oooo.2015.09.004 26679357

[71] Maurya V Srivastava A Mishra J Gaind R Marak RS Tripathi AK. Oropharyngeal candidiasis and candida colonization in HIV positive patients in northern India. J Infect Dev Ctries (2013) 7(8):608–13. doi: 10.3855/jidc.2801 23949296

[72] Schoofs AG Odds FC Colebunders R Ieven M Goossens H. Cross-sectional study of oral candida carriage in a human immunodeficiency virus (HIV)-seropositive population: predisposing factors, epidemiology and antifungal susceptibility. Mycoses (1998) 41(5-6):203–11. doi: 10.1111/j.1439-0507.1998.tb00325.x 9715634

[73] Aboualigalehdari E Tahmasebi Birgani M Fatahinia M Hosseinzadeh M. Oral colonization by candida species and associated factors in HIV-infected patients in ahvaz, southwest Iran. Epidemiol Health (2020) 42:e2020033. doi: 10.4178/epih.e2020033 32512666 PMC7644944

[74] Kirti YK. Prevalence of oral candidiasis in Indian HIV sero-positive patients with CD4(+) cell count correlation. Indian J Otolaryngol Head Neck Surg (2019) 71(1):124–7. doi: 10.1007/s12070-018-1342-3 PMC6401036 30906728

[75] Heron SE Elahi S. HIV Infection and compromised mucosal immunity: Oral manifestations and systemic inflammation. Front Immunol (2017) 8:241. doi: 10.3389/fimmu.2017.00241 28326084 PMC5339276

[76] Li Y Saxena D Chen Z Liu G Abrams WR Phelan JA. HIV Infection and microbial diversity in saliva. J Clin Microbiol (2014) 52(5):1400–11. doi: 10.1128/JCM.02954-13 PMC3993673 24523469

[77] Huang YS Yang JJ Lee NY Chen GJ Ko WC Sun HY. Treatment of pneumocystis jirovecii pneumonia in HIV-infected patients: a review. Expert Rev Anti Infect Ther (2017) 15(9):873–92. doi: 10.1080/14787210.2017.1364991 28782390

[78] Hitzenbichler F Mohr A Salzberger B. [Pneumocystis jirovecii pneumonia-an opportunistic infection undergoing change]. Internist (Berl) (2019) 60(7):669–77. doi: 10.1007/s00108-019-0616-5 31089770

[79] Shibata S Kikuchi T. Pneumocystis pneumonia in HIV-1-infected patients. Respir Investig (2019) 57(3):213–9. doi: 10.1016/j.resinv.2019.01.009 30824356

[80] Buchacz K Baker RK Palella FJ Jr. Chmiel JS Lichtenstein KA Novak RM. AIDS-defining opportunistic illnesses in US patients, 1994-2007: a cohort study. AIDS (2010) 24(10):1549–59. doi: 10.1097/QAD.0b013e32833a3967 20502317

[81] Limper AH Adenis A Le T Harrison TS. Fungal infections in HIV/AIDS. Lancet Infect Dis (2017) 17(11):e334–43. doi: 10.1016/S1473-3099(17)30303-1 28774701

[82] Brown GD Denning DW Gow NA Levitz SM Netea MG White TC. Hidden killers: human fungal infections. Sci Transl Med (2012) 4(165):165rv113. doi: 10.1126/scitranslmed.3004404 23253612

[83] Bongomin F Gago S Oladele RO Denning DW. Global and multi-national prevalence of fungal diseases-estimate precision. J Fungi (Basel) (2017) 3(4):57. doi: 10.3390/jof3040057 29371573 PMC5753159

[84] Bozorgomid A Hamzavi Y Heidari Khayat S Mahdavian B Bashiri H. Pneumocystis jirovecii pneumonia and human immunodeficiency virus Co-infection in Western Iran. Iran J Public Health (2019) 48(11):2065–9. PMC6961188 31970106

[85] Cilloniz C Dominedo C Alvarez-Martinez MJ Moreno A Garcia F Torres A. Pneumocystis pneumonia in the twenty-first century: HIV-infected versus HIV-uninfected patients. Expert Rev Anti Infect Ther (2019) 17(10):787–801. doi: 10.1080/14787210.2019.1671823 31550942

[86] Huang L Cattamanchi A Davis JL den Boon S Kovacs J Meshnick S. HIV-Associated pneumocystis pneumonia. Proc Am Thorac Soc (2011) 8(3):294–300. doi: 10.1513/pats.201009-062WR 21653531 PMC3132788

[87] Kelly MN Shellito JE. Current understanding of pneumocystis immunology. Future Microbiol (2010) 5(1):43–65. doi: 10.2217/fmb.09.116 20020829 PMC3702169

[88] Carmona EM Vassallo R Vuk-Pavlovic Z Standing JE Kottom TJ Limper AH. Pneumocystis cell wall beta-glucans induce dendritic cell costimulatory molecule expression and inflammatory activation through a fas-fas ligand mechanism. J Immunol (2006) 177(1):459–67. doi: 10.4049/jimmunol.177.1.459 16785543

[89] Carmona EM Kottom TJ Hebrink DM Moua T Singh RD Pagano RE. Glycosphingolipids mediate pneumocystis cell wall beta-glucan activation of the IL-23/IL-17 axis in human dendritic cells. Am J Respir Cell Mol Biol (2012) 47(1):50–9. doi: 10.1165/rcmb.2011-0159OC PMC3402796 22343219

[90] Otieno-Odhiambo P Wasserman S Hoving JC. The contribution of host cells to pneumocystis immunity: An update. Pathogens (2019) 8(2):52. doi: 10.3390/pathogens8020052 31010170 PMC6631015

[91] Rajasingham R Smith RM Park BJ Jarvis JN Govender NP Chiller TM. Global burden of disease of HIV-associated cryptococcal meningitis: an updated analysis. Lancet Infect Dis (2017) 17(8):873–81. doi: 10.1016/S1473-3099(17)30243-8 PMC5818156 28483415

[92] Veltman JA Bristow CC Klausner JD. Meningitis in HIV-positive patients in sub-Saharan Africa: a review. J Int AIDS Soc (2014) 17:19184. doi: 10.7448/IAS.17.1.19184 25308903 PMC4195174

[93] Loyse A Thangaraj H Easterbrook P Ford N Roy M Chiller T. Cryptococcal meningitis: improving access to essential antifungal medicines in resource-poor countries. Lancet Infect Dis (2013) 13(7):629–37. doi: 10.1016/S1473-3099(13)70078-1 23735626

[94] Loyse A Burry J Cohn J Ford N Chiller T Ribeiro I. Leave no one behind: response to new evidence and guidelines for the management of cryptococcal meningitis in low-income and middle-income countries. Lancet Infect Dis (2019) 19(4):e143–7. doi: 10.1016/S1473-3099(18)30493-6 30344084

[95] Perfect JR Dismukes WE Dromer F Goldman DL Graybill JR Hamill RJ. Clinical practice guidelines for the management of cryptococcal disease: 2010 update by the infectious diseases society of america. Clin Infect Dis (2010) 50(3):291–322. doi: 10.1086/649858 20047480 PMC5826644

[96] Neal LM Xing E Xu J Kolbe JL Osterholzer JJ Segal BM. CD4(+) T cells orchestrate lethal immune pathology despite fungal clearance during cryptococcus neoformans meningoencephalitis. mBio (2017) 8(6):e01415-17. doi: 10.1128/mBio.01415-17 29162707 PMC5698549

[97] Pirofski LA Casadevall A. Immune-mediated damage completes the parabola: Cryptococcus neoformans pathogenesis can reflect the outcome of a weak or strong immune response. mBio (2017) 8(6):e02063-17. doi: 10.1128/mBio.02063-17 PMC5727418 29233901

[98] Perez-Jacoiste Asin MA Bisbal O Iribarren JA Perez-Rivilla A Mican R Dronda F. Cryptococcal infection in HIV-infected patients with CD4(+) T-cell counts under 100/muL diagnosed in a high-income country: a multicentre cohort study. Clin Microbiol Infect (2021) 27(8):1171 e1171–1171 e1177. doi: 10.1016/j.cmi.2020.09.053 33069858

[99] Iyer KR Revie NM Fu C Robbins N Cowen LE. Treatment strategies for cryptococcal infection: challenges, advances and future outlook. Nat Rev Microbiol (2021) 19(7):454–66. doi: 10.1038/s41579-021-00511-0 PMC7868659 33558691

[100] Li A Zhu W Yin J Huang X Sun L Hua W. A preliminary study on the characteristics of Th1/Th2 immune response in cerebrospinal fluid of AIDS patients with cryptococcal meningitis. BMC Infect Dis (2021) 21(1):500. doi: 10.1186/s12879-021-06138-z 34051748 PMC8164222

[101] Jarvis JN Bicanic T Loyse A Namarika D Jackson A Nussbaum JC. Determinants of mortality in a combined cohort of 501 patients with HIV-associated cryptococcal meningitis: implications for improving outcomes. Clin Infect Dis (2014) 58(5):736–45. doi: 10.1093/cid/cit794 PMC3922213 24319084

[102] Jarvis JN Meintjes G Rebe K Williams GN Bicanic T Williams A. Adjunctive interferon-gamma immunotherapy for the treatment of HIV-associated cryptococcal meningitis: a randomized controlled trial. AIDS (2012) 26(9):1105–13. doi: 10.1097/QAD.0b013e3283536a93 PMC3640254 22421244

[103] Boulware DR Meya DB Bergemann TL Wiesner DL Rhein J Musubire A. Clinical features and serum biomarkers in HIV immune reconstitution inflammatory syndrome after cryptococcal meningitis: a prospective cohort study. PLos Med (2010) 7(12):e1000384. doi: 10.1371/journal.pmed.1000384 21253011 PMC3014618

[104] Narayanasamy S Dat VQ Thanh NT Ly VT Chan JF Yuen KY. A global call for talaromycosis to be recognised as a neglected tropical disease. Lancet Glob Health (2021) 9(11):e1618–22. doi: 10.1016/S2214-109X(21)00350-8 PMC10014038 34678201

[105] Qin Y Huang X Chen H Liu X Li Y Hou J. Burden of talaromyces marneffei infection in people living with HIV/AIDS in Asia during ART era: a systematic review and meta-analysis. BMC Infect Dis (2020) 20(1):551. doi: 10.1186/s12879-020-05260-8 32727383 PMC7392840

[106] Jiang J Meng S Huang S Ruan Y Lu X Li JZ. Effects of talaromyces marneffei infection on mortality of HIV/AIDS patients in southern China: a retrospective cohort study. Clin Microbiol Infect (2019) 25(2):233–41. doi: 10.1016/j.cmi.2018.04.018 29698815

[107] Pruksaphon K Nosanchuk JD Ratanabanangkoon K Youngchim S. Talaromyces marneffei infection: Virulence, intracellular lifestyle and host defense mechanisms. J Fungi (Basel) (2022) 8(2):200. doi: 10.3390/jof8020200 35205954 PMC8880324

[108] Srinoulprasert Y Pongtanalert P Chawengkirttikul R Chaiyaroj SC. Engagement of penicillium marneffei conidia with multiple pattern recognition receptors on human monocytes. Microbiol Immunol (2009) 53(3):162–72. doi: 10.1111/j.1348-0421.2008.00102.x 19302527

[109] Pongpom M Vanittanakom P Nimmanee P Cooper CR Jr. Vanittanakom N. Adaptation to macrophage killing by talaromyces marneffei. Future Sci OA (2017) 3(3):FSO215. doi: 10.4155/fsoa-2017-0032 28884011 PMC5583664

[110] Lu S Hu Y Lu C Zhang J Li X Xi L. Development of *in vitro* macrophage system to evaluate phagocytosis and intracellular fate of penicillium marneffei conidia. Mycopathologia (2013) 176(1-2):11–22. doi: 10.1007/s11046-013-9650-3 23645133

[111] Ellett F Pazhakh V Pase L Benard EL Weerasinghe H Azabdaftari D. Macrophages protect talaromyces marneffei conidia from myeloperoxidase-dependent neutrophil fungicidal activity during infection establishment in vivo. PLos Pathog (2018) 14(6):e1007063. doi: 10.1371/journal.ppat.1007063 29883484 PMC6010348

[112] Tang Y Zhang H Xu H Zeng W Qiu Y Tan C. Dendritic cells promote treg expansion but not Th17 generation in response to talaromyces marneffei yeast cells. Infect Drug Resist (2020) 13:805–13. doi: 10.2147/IDR.S239906 PMC7075240 32210595

[113] Wang M Li L Xiao S Chen W Hu F Li F. The association of TLR2, TLR3, and TLR9 gene polymorphisms with susceptibility to talaromycosis among han Chinese AIDS patients in guangdong. Front Cell Infect Microbiol (2021) 11:625461. doi: 10.3389/fcimb.2021.625461 33777838 PMC7991721

[114] Guo J Ning XQ Ding JY Zheng YQ Shi NN Wu FY. Anti-IFN-gamma autoantibodies underlie disseminated talaromyces marneffei infections. J Exp Med (2020) 217(12):e20190502. doi: 10.1084/jem.20190502 32880631 PMC7953730

[115] Narayanasamy S Dougherty J van Doorn HR Le T. Pulmonary talaromycosis: A window into the immunopathogenesis of an endemic mycosis. Mycopathologia (2021) 186(5):707–15. doi: 10.1007/s11046-021-00570-0 PMC8536569 34228343

[116] Manion M Boulougoura A Naqvi N Lage SL Richards E Grivas C. Polyfunctional antigen specific CD4+/- T cell responses in patients with HIV/AIDS and histoplasmosis immune reconstitution inflammatory syndrome. Clin Infect Dis (2022) ciac514. doi: 10.1093/cid/ciac514 35767272 PMC10169433

[117] Chen H Yuan Q Hu H Wang J Yu M Yang Q. Hemophagocytic lymphohistiocytosis secondary to disseminated histoplasmosis in HIV seronegative patients: A case report and review of the literature. Front Cell Infect Microbiol (2022) 12:847950. doi: 10.3389/fcimb.2022.847950 35782129 PMC9245433

[118] Wheat LJ Azar MM Bahr NC Spec A Relich RF Hage C. Histoplasmosis. Infect Dis Clin North Am (2016) 30(1):207–27. doi: 10.1016/j.idc.2015.10.009 26897068

[119] Adenis AA Valdes A Cropet C McCotter OZ Derado G Couppie P. Burden of HIV-associated histoplasmosis compared with tuberculosis in Latin America: a modelling study. Lancet Infect Dis (2018) 18(10):1150–9. doi: 10.1016/S1473-3099(18)30354-2 PMC6746313 30146320

[120] Basso RP Poester VR Benelli JL Stevens DA Xavier MO. Disseminated histoplasmosis in persons with HIV/AIDS, southern Brazil, 2010-2019. Emerg Infect Dis (2022) 28(3):721–4. doi: 10.3201/eid2803.212150 PMC8888216 35202533

[121] Tobon AM Gomez BL. Pulmonary histoplasmosis. Mycopathologia (2021) 186(5):697–705. doi: 10.1007/s11046-021-00588-4 34498137

[122] Garfoot AL Rappleye CA. Histoplasma capsulatum surmounts obstacles to intracellular pathogenesis. FEBS J (2016) 283(4):619–33. doi: 10.1111/febs.13389 PMC4827932 26235362

[123] Almeida MA Almeida-Paes R Guimaraes AJ Valente RH Soares CMA Zancope-Oliveira RM. Immunoproteomics reveals pathogen's antigens involved in homo sapiens-histoplasma capsulatum interaction and specific linear b-cell epitopes in histoplasmosis. Front Cell Infect Microbiol (2020) 10:591121. doi: 10.3389/fcimb.2020.591121 33251160 PMC7673445

[124] Holding KJ Dworkin MS Wan PC Hanson DL Klevens RM Jones JL. Aspergillosis among people infected with human immunodeficiency virus: incidence and survival. Adult Adolesc Spectr HIV Dis Project Clin Infect Dis (2000) 31(5):1253–7. doi: 10.1086/317452 11073760

[125] Denis B Guiguet M de Castro N Mechai F Revest M Melica G. Relevance of EORTC criteria for the diagnosis of invasive aspergillosis in HIV-infected patients, and survival trends over a 20-year period in France. Clin Infect Dis (2015) 61(8):1273–80. doi: 10.1093/cid/civ492 26123932

[126] Scriven JE Tenforde MW Levitz SM Jarvis JN. Modulating host immune responses to fight invasive fungal infections. Curr Opin Microbiol (2017) 40:95–103. doi: 10.1016/j.mib.2017.10.018 29154044 PMC5816974

[127] Ward RA Vyas JM. The first line of defense: effector pathways of anti-fungal innate immunity. Curr Opin Microbiol (2020) 58:160–5. doi: 10.1016/j.mib.2020.10.003 PMC7746574 33217703

[128] Kumar V van de Veerdonk FL Netea MG. Antifungal immune responses: emerging host-pathogen interactions and translational implications. Genome Med (2018) 10(1):39. doi: 10.1186/s13073-018-0553-2 29801518 PMC5968547

[129] Underhill DM Pearlman E. Immune interactions with pathogenic and commensal fungi: A two-way Street. Immunity (2015) 43(5):845–58. doi: 10.1016/j.immuni.2015.10.023 PMC4865256 26588778

[130] Bartemes KR Kita H. Innate and adaptive immune responses to fungi in the airway. J Allergy Clin Immunol (2018) 142(2):353–63. doi: 10.1016/j.jaci.2018.06.015 PMC6083885 30080527

[131] Desai JV Lionakis MS. The role of neutrophils in host defense against invasive fungal infections. Curr Clin Microbiol Rep (2018) 5(3):181–9. doi: 10.1007/s40588-018-0098-6 PMC6758935 31552161

[132] Wang Y Pawar S Dutta O Wang K Rivera A Xue C. Macrophage mediated immunomodulation during cryptococcus pulmonary infection. Front Cell Infect Microbiol (2022) 12:859049. doi: 10.3389/fcimb.2022.859049 35402316 PMC8987709

[133] Hunniger K Kurzai O. Phagocytes as central players in the defence against invasive fungal infection. Semin Cell Dev Biol (2019) 89:3–15. doi: 10.1016/j.semcdb.2018.03.021 29601862

[134] Casulli S Elbim C. Interactions between human immunodeficiency virus type 1 and polymorphonuclear neutrophils. J Innate Immun (2014) 6(1):13–20. doi: 10.1159/000353588 23867213 PMC6741617

[135] Musubire AK Meya DB Rhein J Meintjes G Bohjanen PR Nuwagira E. Blood neutrophil counts in HIV-infected patients with cryptococcal meningitis: Association with mortality. PLos One (2018) 13(12):e0209337. doi: 10.1371/journal.pone.0209337 30596708 PMC6312212

[136] Kalem MC Humby MS Wohlfert EA Jacobs A Panepinto JC. Cryptococcus neoformans coinfection dampens the TNF-alpha response in HIV-1-Infected human THP-1 macrophages. mSphere (2021) 6(2):e00213-21. doi: 10.1128/mSphere.00213-21 33762317 PMC8546698

[137] Lubow J Collins KL. Vpr is a VIP: HIV vpr and infected macrophages promote viral pathogenesis. Viruses (2020) 12(8):809. doi: 10.3390/v12080809 32726944 PMC7472745

[138] Koziel H Eichbaum Q Kruskal BA Pinkston P Rogers RA Armstrong MY. Reduced binding and phagocytosis of pneumocystis carinii by alveolar macrophages from persons infected with HIV-1 correlates with mannose receptor downregulation. J Clin Invest (1998) 102(7):1332–44. doi: 10.1172/JCI560 PMC508980 9769325

[139] Crowe SM Vardaxis NJ Kent SJ Maerz AL Hewish MJ McGrath MS. HIV Infection of monocyte-derived macrophages *in vitro* reduces phagocytosis of candida albicans. J Leukoc Biol (1994) 56(3):318–27. doi: 10.1002/jlb.56.3.318 8083603

[140] Roilides E Holmes A Blake C Pizzo PA Walsh TJ. Defective antifungal activity of monocyte-derived macrophages from human immunodeficiency virus-infected children against aspergillus fumigatus. J Infect Dis (1993) 168(6):1562–5. doi: 10.1093/infdis/168.6.1562 8245547

[141] Maldonado S Fitzgerald-Bocarsly P. Antifungal activity of plasmacytoid dendritic cells and the impact of chronic HIV infection. Front Immunol (2017) 8:1705. doi: 10.3389/fimmu.2017.01705 29255464 PMC5723005

[142] Miller E Bhardwaj N. Dendritic cell dysregulation during HIV-1 infection. Immunol Rev (2013) 254(1):170–89. doi: 10.1111/imr.12082 PMC5590719 23772620

[143] Boasso A Herbeuval JP Hardy AW Anderson SA Dolan MJ Fuchs D. HIV Inhibits CD4+ T-cell proliferation by inducing indoleamine 2,3-dioxygenase in plasmacytoid dendritic cells. Blood (2007) 109(8):3351–9. doi: 10.1182/blood-2006-07-034785 PMC1852248 17158233

[144] Fernandez-Garcia OA Cuellar-Rodriguez JM. Immunology of fungal infections. Infect Dis Clin North Am (2021) 35(2):373–88. doi: 10.1016/j.idc.2021.03.006 34016282

[145] Speakman EA Dambuza IM Salazar F Brown GD. T Cell antifungal immunity and the role of c-type lectin receptors. Trends Immunol (2020) 41(1):61–76. doi: 10.1016/j.it.2019.11.007 31813764 PMC7427322

[146] Verma A Wuthrich M Deepe G Klein B. Adaptive immunity to fungi. Cold Spring Harb Perspect Med (2014) 5(3):a019612. doi: 10.1101/cshperspect.a019612 25377140 PMC4355251

[147] Scheffold A Bacher P LeibundGut-Landmann S. T Cell immunity to commensal fungi. Curr Opin Microbiol (2020) 58:116–23. doi: 10.1016/j.mib.2020.09.008 33120172

[148] Pathakumari B Liang G Liu W. Immune defence to invasive fungal infections: A comprehensive review. BioMed Pharmacother (2020) 130:110550. doi: 10.1016/j.biopha.2020.110550 32739740

[149] Rohatgi S Pirofski LA. Host immunity to cryptococcus neoformans. Future Microbiol (2015) 10(4):565–81. doi: 10.2217/fmb.14.132 PMC4523559 25865194

[150] Wich M Greim S Ferreira-Gomes M Kruger T Kniemeyer O Brakhage AA. Functionality of the human antibody response to candida albicans. Virulence (2021) 12(1):3137–48. doi: 10.1080/21505594.2021.2015116 PMC8923069 34923920

[151] Xu L Guo Y Zhao Y Xu Y Peng X Yang Z. Chemokine and cytokine cascade caused by skewing of the Th1-Th2 balance is associated with high intracranial pressure in HIV-associated cryptococcal meningitis. Mediators Inflammation (2019) 2019:2053958. doi: 10.1155/2019/2053958 PMC7012228 32082071

[152] Ayatollahi Mousavi SA Asadikaram G Nakhaee N Izadi A. Plasma levels of IFN-gamma, IL-4, IL-6 and IL-17 in HIV-positive patients with oral candidiasis. Jundishapur J Microbiol (2016) 9(2):e32021. doi: 10.5812/jjm.32021 27127595 PMC4842255

[153] Jarvis JN Meintjes G Bicanic T Buffa V Hogan L Mo S. Cerebrospinal fluid cytokine profiles predict risk of early mortality and immune reconstitution inflammatory syndrome in HIV-associated cryptococcal meningitis. PLos Pathog (2015) 11(4):e1004754. doi: 10.1371/journal.ppat.1004754 25853653 PMC4390200

[154] Renault C Veyrenche N Mennechet F Bedin AS Routy JP Van de Perre P. Th17 CD4+ T-cell as a preferential target for HIV reservoirs. Front Immunol (2022) 13:822576. doi: 10.3389/fimmu.2022.822576 35197986 PMC8858966

[155] Wacleche VS Landay A Routy JP Ancuta P. The Th17 lineage: From barrier surfaces homeostasis to autoimmunity, cancer, and HIV-1 pathogenesis. Viruses (2017) 9(10):303. doi: 10.3390/v9100303 29048384 PMC5691654

[156] Liu F Fan X Auclair S Ferguson M Sun J Soong L. Sequential dysfunction and progressive depletion of candida albicans-specific CD4 T cell response in HIV-1 infection. PLos Pathog (2016) 12(6):e1005663. doi: 10.1371/journal.ppat.1005663 27280548 PMC4900544

[157] Zhang Q Frange P Blanche S Casanova JL. Pathogenesis of infections in HIV-infected individuals: insights from primary immunodeficiencies. Curr Opin Immunol (2017) 48:122–33. doi: 10.1016/j.coi.2017.09.002 PMC5682227 28992464

[158] Ruggiero A Pascucci GR Cotugno N Dominguez-Rodriguez S Rinaldi S Tagarro A. Determinants of b-cell compartment hyperactivation in European adolescents living with perinatally acquired HIV-1 after over 10 years of suppressive therapy. Front Immunol (2022) 13:860418. doi: 10.3389/fimmu.2022.860418 35432380 PMC9009387

[159] Okurut S Meya DB Bwanga F Olobo J Eller MA Cham-Jallow F. B cell compartmentalization in blood and cerebrospinal fluid of HIV-infected ugandans with cryptococcal meningitis. Infect Immun (2020) 88(3):e00779-19. doi: 10.1128/IAI.00779-19 31871098 PMC7035924

[160] Rohatgi S Nakouzi A Carreno LJ Slosar-Cheah M Kuniholm MH Wang T. Antibody and b cell subset perturbations in human immunodeficiency virus-uninfected patients with cryptococcosis. Open Forum Infect Dis (2018) 5(1):ofx255. doi: 10.1093/ofid/ofx255 29354657 PMC5767948

[161] Yoon HA Nakouzi A Chang CC Kuniholm MH Carreno LJ Wang T. Association between plasma antibody responses and risk for cryptococcus-associated immune reconstitution inflammatory syndrome. J Infect Dis (2019) 219(3):420–8. doi: 10.1093/infdis/jiy447 PMC6325352 30010905

[162] Blount RJ Daly KR Fong S Chang E Grieco K Greene M. Effects of clinical and environmental factors on bronchoalveolar antibody responses to pneumocystis jirovecii: A prospective cohort study of HIV+ patients. PLos One (2017) 12(7):e0180212. doi: 10.1371/journal.pone.0180212 28692651 PMC5503245

[163] Blount RJ Jarlsberg LG Daly KR Worodria W Davis JL Cattamanchi A. Serologic responses to recombinant pneumocystis jirovecii major surface glycoprotein among Ugandan patients with respiratory symptoms. PLos One (2012) 7(12):e51545. doi: 10.1371/journal.pone.0051545 23284710 PMC3528778

